# Case report: SARS-CoV-2 infection as a trigger for diabetic ketoacidosis and newly detected pancreatic autoantibodies

**DOI:** 10.3389/fendo.2022.983206

**Published:** 2022-08-10

**Authors:** Rahul Mishra, Ghada Elshimy, Lakshmi Kannan, Aasems Jacob, Rishi Raj

**Affiliations:** ^1^ Department of Hematology and Oncology, Cleveland Clinic Foundation, Cleveland, OH, United States; ^2^ Department of Endocrinology, Diabetes, and Metabolism, Medical College of Georgia, Augusta University, Augusta, GA, United States; ^3^ Department of Nephrology, Pikeville Medical Center, Pikeville, KY, United States; ^4^ Department of Nephrology, Kentucky College of Osteopathic Medicine, University of Pikeville, Pikeville, KY, United States; ^5^ Department of Hematology and Oncology, Pikeville Medical Center, Pikeville, KY, United States; ^6^ Department of Hematology and Oncology, Kentucky College of Osteopathic Medicine, University of Pikeville, Pikeville, KY, United States; ^7^ Department of Endocrinology, Diabetes, and Metabolism, Pikeville Medical Center, Pikeville, KY, United States; ^8^ Department of Endocrinology, Diabetes, and Metabolism, Kentucky College of Osteopathic Medicine, University of Pikeville, Pikeville, KY, United States

**Keywords:** COVID – 19, SARS – CoV – 2, type 1 diabetes (T1D), autoantibodies, autoimminity

## Abstract

A 39-year-old-woman with a past medical history of type 2 diabetes mellitus (T2DM) on oral hypoglycemic agents presented to the emergency room with nausea, vomiting, shortness of breath, and altered mental status. Seven days prior to presentation, she was diagnosed with severe acute respiratory syndrome coronavirus 2 (SARS-CoV-2) infection. Laboratory workup on presentation confirmed the diagnosis of diabetic ketoacidosis (DKA) (blood glucose 523 mg/dl, beta-hydroxybutyrate 8.91 mmol/l, pH 6.9, bicarbonate 11 mEq/l, anion gap 25 mEq/l, and HbA1c 10.8%). She was managed for DKA with hydration and insulin drip and discharged home. However, to our surprise, at the 2-week follow-up visit, she was found to have positive antibodies for zinc transporter 8 (ZnT8) (samples were collected on day of presentation). The rest of her antibodies associated with T1DM were negative. She was therefore started on a basal-bolus regimen and managed as type 1 diabetes mellitus (T1DM). Our case illustrates that there is an increased risk of T1DM following infection with SARS-CoV-2.

## Introduction

COVID-19 is caused by SARS-CoV-2 infection and can have severe adverse outcomes, including death (1). The endogenous receptor of the virus, angiotensin-converting enzyme-2 (ACE2), enables the virus to infect other organs including the endocrine system in addition to the lungs. Infection with SARS-CoV-2 is known to act as an infectious trigger for autoimmune conditions such as Guillain–Barré syndrome, systemic lupus erythematosus, thrombotic thrombocytopenic purpura, autoimmune hemolytic anemia, thrombocytopenic purpura, and autoimmune thyroid disease (2, 3). Multicentric studies have confirmed the increased prevalence of new-onset T1DM during the current COVID-19 pandemic (4, 5). Although this increased incidence of T1DM following COVID-19 is well established in epidemiological studies, the current literature lacks data on its pathogenesis. The majority of the published studies are retrospective with significant heterogeneity among them in terms of the study population. Moreover, many of these population studies lack data on immunologic workup, essential to understanding the pathogenesis of T1DM following COVID-19. Hence, there is a gap in our understanding of how infection with SARS-COV-2 may be associated with the development of antibodies against beta-cells, ultimately leading to T1DM. With our case, we want to provide a possible insight into the critical pathophysiologic aspect of the development of T1DM among patients following COVID-19.

## Case report

A 39-year-old-woman with a past medical history of T2DM (diagnosed 1.5 years ago prior to presentation and managed with metformin and dapagliflozin) presented to the emergency room with chief complaints of nausea, vomiting, shortness of breath, and altered mental status in March 2022. Seven days prior to presentation, she tested positive for COVID-19 and was in home quarantine. Regarding her history of diabetes, she was diagnosed with type 2 diabetes based on her HbA1c level of 6.6 in 2020, and since then her HbA1c level has ranged between 6.1 and 9.3. On presentation, she had tachycardia with a pulse rate of 115 beats per minute. The rest of her vitals were stable with blood pressure of 109/68 mmHg, saturation of 97% on room air, and temperature of 98.8 F. BMI was 32 kg/m^2^. Laboratory workup confirmed the diagnosis of diabetic ketoacidosis (DKA) (blood glucose 523 mg/dl, beta-hydroxybutyrate 8.91 mmol/l, pH 6.9, bicarbonate 11 mEq/l, anion gap 25 mEq/l, potassium 2.9 mEq/l, and HbA1c 10.8%). Rapid nasopharyngeal testing showed a negative result for other common viral etiologies including adenovirus, influenza A/B, parainfluenza, and enterovirus. The complete laboratory workup at presentation is summarized in **Table 1**. Chest x-ray showed unremarkable results without infiltrates. Considering diagnosis of DKA, she was treated with aggressive intravenous hydration, potassium replacement, and insulin drip per DKA protocol. She was also treated with intravenous bicarbonate. Her hyperglycemia was controlled after 2 days, following which the basal-bolus insulin regimen was initiated. She was discharged in a reasonably comfortable condition on dapagliflozin 10 mg daily, metformin 500 mg twice daily, and premix insulin 70/30 (insulin aspart protamine and insulin aspart) 10 units twice a day before meals. At a 2-week follow-up, her serology results (that were collected on day of presentation) came back positive for zinc transporter 8 (ZnT8) antibodies (67 U/ml; reference range <15 U/ml) and negative for glutamic acid decarboxylase 65 (GAD65) antibodies, insulinoma-associated protein 2 (IA2) antibodies, islet cell antibodies (ICA), and anti-insulin antibodies. Hence, metformin and dapagliflozin were discontinued and diabetes treatment was switched to insulin-only (insulin glargine 20 units daily and insulin lispro 5 units three times a day before meals) regimen with a continuous glucose monitoring device for management as T1DM.

**Table 1:** Laboratory investigation on admission.

Blood investigations (normal range, units)	Values
Blood glucose (random, <200 mg/dl)	523
Sodium (133-144, mEq/L)	137
WBC (3-11.3, K/µL)	13.26
Hemoglobin (10-16, gm/dL)	16.1
Platelet count (182-369, K/µL)	290
Potassium (3.6-5.2 mEq/L)	2.9
Calcium (8.5-10.1, mg/dL)	9.1
Chloride (98-107, mmol/L)	99
Bicarbonate (22-29 mEq/L)	12
Blood urea nitrogen (7-18, mg/dL)	15
Creatinine (0.60-1.30, mg/dL)	1.5
Estimated glomerular filtration rate (60-200, mL/min/m^2^)	61.7
Albumin (3.4-5.0, g/dL)	2.5
Alanine transaminase (12-78, U/L)	9
Aspartate transaminase (15-37, U/L)	6
Amylase (25-115, U/L)	86
Lipase (73-393, U/L)	483
Creatinine kinase (21-232, U/L)	49
Troponin I (0-53.7, ng/L)	16.3
Lactic acid (0.4-2, mmol/L)	1.1
Beta hydroxybutyrate (0.02-0.27, mmol/L)	8.91
Hemoglobin A1c (<5.6, %)	10.8
**Urinary investigation**	
Bacteria (absent, count/HPF)	Trace
Glucose (negative, mg/dL)	4+
Ketone (negative, mg/dL)	4+
Blood (negative, NA)	3+
Protein (negative, mg/dL)	2+
WBC (0-2, mg/dL)	0-2

## Discussion

Acute hyperglycemic states (such as DKA and hyperosmolar hyperglycemia) among patients with diabetes can have catastrophic outcomes. Although DKA is a telltale complication of T1DM, it is not uncommon among patients with T2DM, particularly during insulinopenic states (6, 7). Patients with poorly controlled diabetes (such as our patient with HbA1c of 10.8%) are at a higher risk of DKA in the setting of the acute infection (8). Contrastingly, some studies reported that patients with diabetes who developed DKA following SARS-CoV-2 infection had good glycemic control prior to the hospitalization (9). Our patient had relatively new-onset diabetes (diagnosed within 1.5 years) and presented with DKA following COVID-19 infection, demonstrating a temporal association between onset of T1DM (evidenced by presentation in DKA, and need for insulin) following infection with SARS-CoV-2. Prior to this episode, the patient was treated with oral hypoglycemic agents for T2DM and never had a DKA. Interestingly, the antibodies against ZnT8 were detected upon follow-up visit confirming the diagnosis of T1DM. ZnT8 is a well-known autoantigen leading to the development of T1DM and can be detectable in up to 80% of patients with T1DM. Furthermore, it can be the only detectable autoantibody in approximately 26% of patients with antibody-negative (insulin, GAD, IA-2, and ICA) T1DM (10).

Various studies have demonstrated an increased incidence of newly diagnosed T1DM during the COVID-19 pandemic, compared to the pre-pandemic era (5). In a retrospective study by experts from the Center for Disease Control and Prevention (CDC), an analysis of two databases (IQVIA and HealthVerity) revealed a higher incidence of newly diagnosed diabetes (166% and 33% in IQVIA and HealthVerity databases, respectively) following COVID-19 (4). Besides increased incidence of T1DM, there is increased frequency and severity of DKA at the time of T1DM diagnosis during the current pandemic. Although the increased incidence of T1DM following COVID-19 is well established in epidemiological studies, the current literature still has a paucity of data on its etiopathogenesis with the majority of the published literature being retrospective, with significant heterogeneity, and with a lack of data on immunologic workup.

Multiple studies have hypothesized COVID-19 as a downstream precipitator of autoantibody-positive T1DM, with a possible inciting autoimmune trigger a while ago (11). Some studies have reported virus-mediated apoptosis of beta cells (pancreatic islet) in COVID-19 which has signaling pathways similar to those seen in T1DM patients (12). A stem cell study suggests that the SARS-CoV-2 virus can enter pancreatic islet cells *via* the ACE2 receptor leading to β-cell damage and a new-onset diabetes (13). While our report does not prove a link, we postulate that SARS-CoV-2 exposure could have potentially contributed to the observed effect in our case leading to precipitation of T1DM. Recently, a similar report of autoantibody (GAD and ZnT8)-positive T1DM, following an asymptomatic SARS-CoV-2 (confirmed with molecular testing), was described in a pediatric patient, in which authors postulated SARS-CoV-2 infection to be the precipitating event for T1DM (11).

One limitation of our article is that it lacks information on the variant type of SARS-CoV-2, which led to precipitation of DKA and positive antibodies, which could have helped understand pathogenesis. Nonetheless, our case supports the association between infection with SARS-CoV-2 and the development of pancreatic autoantibodies, adding evidence for the adult population. Although our patient did not have baseline autoantibodies assays, the long-term follow-up of the patient would be interesting to check if the patient develops more diabetes-related autoantibodies in due course. This is an interesting and evolving field needing more studies investigating pancreatic antibody levels in patients with newly diagnosed T1DM following COVID-19 to better understand this phenomenon.

## Data availability statement

The original contributions presented in the study are included in the article/supplementary material. Further inquiries can be directed to the corresponding authors.

## Ethics statement

Ethical review and approval was not required for the study on human participants in accordance with the local legislation and institutional requirements. The patients/participants provided their written informed consent to participate in this study. Written informed consent was obtained from the individual(s) for the publication of any potentially identifiable images or data included in this article.

## Author contributions

RM and RR gathered patient data. RM, GE, LK, AJ, and RR contributed to the literature review and drafting of the manuscript. All authors approved the manuscript in its entirety.

## Conflict of interest

The authors declare that the research was conducted in the absence of any commercial or financial relationships that could be construed as a potential conflict of interest.

## Publisher’s note

All claims expressed in this article are solely those of the authors and do not necessarily represent those of their affiliated organizations, or those of the publisher, the editors and the reviewers. Any product that may be evaluated in this article, or claim that may be made by its manufacturer, is not guaranteed or endorsed by the publisher.
